# Artery of Percheron infarction: a case report

**DOI:** 10.1186/s13256-017-1375-3

**Published:** 2017-08-12

**Authors:** Axel Sandvig, Sandra Lundberg, Jiri Neuwirth

**Affiliations:** 1 Department of Pharmacology and Clinical Neurosciences, Division of Neuro, Head and Neck, Umeå University Hospital, Umeå, Sweden; 2 Department of Neuromedicine and Movement Science, Faculty of Medicine and Health Sciences, Norwegian University of Science and Technology, Trondheim, Norway; 3 Department of Radiation Sciences, Umeå University Hospital, Umeå, Sweden

**Keywords:** Artery of Percheron, Infarct, Thalamus, Computed tomography

## Abstract

**Background:**

The artery of Percheron is a rare anatomic variant of arterial supply to the paramedian thalamus and rostral midbrain, and occlusion of the artery of Percheron results in bilateral paramedian thalamic infarcts with or without midbrain involvement. Acute artery of Percheron infarcts represent 0.1 to 2% of total ischemic stroke. However, of thalamic strokes, occlusion of artery of Percheron is the cause in 4 to 35% of cases. Early diagnosis of artery of Percheron infarction can be challenging because it is infrequent and early computed tomography or magnetic resonance imaging may be negative. Thus, it can be confused with other neurological conditions such as tumors and infections.

**Case presentation:**

This is a retrospective case study of a 56-year-old white man admitted to Umeå University Hospital and diagnosed with an artery of Percheron infarction. Medical records and the neuroradiological database were reviewed, and the diagnosis was made based on typical symptoms and radiological findings of artery of Percheron infarction.

We report the case of a 56-year-old man with a history of overconsumption of alcohol who was found in his home unconscious and hypothermic. He had a Reaction Level Scale-85 score of 4. He developed ventricular fibrillation on arrival at our emergency department, and cardiopulmonary resuscitation successfully restored sinus rhythm within an estimated 2 minutes of onset. He was then put on cardiopulmonary bypass for rewarming. The initial head computed tomography performed on admission was wrongly assessed as unremarkable. Bilateral ischemia in the paramedian thalamic nuclei and pons were first documented on a follow-up computed tomography on day 24 after hospitalization. He died on day 35 after hospitalization.

**Conclusions:**

Artery of Percheron infarcts are rare. The radiological diagnosis can initially often be judged as normal and in combination with variability in the neurological symptoms it is a rather difficult condition to diagnose. For these reasons few clinicians have much experience with this type of infarct, which may delay diagnosis and initiation of appropriate treatment.

## Background

The vascular supply of the thalamus is classically divided into four territories: tuberothalamic, inferolateral, paramedian, and posterior choroidal [1]. The paramedian territory is supplied by paramedian arteries, also called thalamoperforating arteries, which arise from proximal segment of the posterior cerebral artery (P1) [1]. Gerard Percheron described four anatomical variants of arterial supply to the paramedian thalami, including the artery of Percheron (AOP), a rare variant of paramedian arterial supply in which a single dominant thalamoperforating artery arises from the P1 and bifurcates to supply both paramedian thalami and, in some cases, the rostral mesencephalon [2–6]. Occlusion of this artery thus results in a characteristic pattern of bilateral paramedian thalamic infarcts with or without mesencephalic infarctions [7–9].

The most common patterns of AOP infarction identified are: bilateral paramedian thalamic with midbrain infarction (43%); bilateral paramedian thalamic infarction only, with no midbrain involvement (38%); and bilateral paramedian thalamic infarction with involvement of anterior thalamus as well as the midbrain (14%) [10].

The exact prevalence of AOP is not known [11, 12]. However, in two autopsy studies, AOP was identified in 11.7% and 7% of the brains investigated [11, 13]. Reports have described that AOP infarcts represent 0.1 to 2.0% of total ischemic stroke [14, 15] and 0.1 to 0.3% of first ever ischemic strokes [14, 16]. Concerning thalamic strokes, occlusion of AOP is the cause in 4 to 35% of cases [3, 10, 17].

The imaging modalities of choice for early diagnosis of AOP infarction are diffusion-weighted imaging (DWI) and fluid-attenuated inversion recovery (FLAIR) [10, 18].

Typical symptoms of bilateral paramedian thalamic infarcts due to occlusion of AOP are vertical gaze palsy, memory impairment, akinetic mutism, confusion, drowsiness, hypersomnolence, or coma [4, 5, 10, 17]. Patients with bilateral paramedian thalamic infarcts accompanied by rostral midbrain lesions also have hemiplegia, cerebellar ataxia, movement dysfunctions, and oculomotor deficits [4, 10].

Treatment of AOP infarction includes thrombolysis and intravenously administered heparin treatment followed by long-term anticoagulants [19]. However, because there often is a delay in diagnosing AOP infarcts, thrombolysis cannot be performed due to its narrow therapeutic window. This emphasizes the importance of early diagnosis so that therapy can be initiated.

The three main factors responsible for delaying AOP infarct diagnosis are: (i) the variety of the presenting neurological symptoms; (ii) the difficulty of diagnosing AOP infarcts in acute computed tomography (CT)/magnetic resonance imaging (MRI) investigations; and (iii) the infrequency of AOP infarcts, which reduces awareness of this condition among physicians [12]. Case presentations are thus a way to address this issue. In this study we report on a patient admitted to Umeå University Hospital, Sweden, diagnosed as having an AOP infarct.

## Case presentation

This is a retrospective case study of a 56-year-old white man diagnosed as having an AOP infarct admitted to Umeå University Hospital, Sweden. The relevant radiology images were obtained from the hospital’s database (PACS) and evaluated by a neuroradiologist as bilateral paramedian thalamic infarcts as a result of an AOP occlusion. The medical records (SYStem Cross) at Umeå University Hospital were accessed and reviewed for his medical history, neurological work-up, and laboratory work-up (ROS). The diagnosis was based on symptoms of AOP infarction as described in the literature, radiological signs of AOP infarction, as well as exclusion of differential diagnoses.

He was taken to our emergency department (ED) after he was found unconscious in his home with open doors and windows. He developed ventricular fibrillation on arrival at our ED. Cardiopulmonary resuscitation (CPR) was immediately initiated and sinus rhythm (SR) was achieved on second defibrillation. He was then put on cardiopulmonary bypass for rewarming. A third ventricular fibrillation then occurred and SR was achieved on first defibrillation within seconds of onset. The total estimated duration of ventricular fibrillation was less than 2 minutes.

His medical history included overconsumption of alcohol. He smoked 20 cigarettes per day. On admission to our hospital there was no information available concerning his medication. On arrival at our ED his body temperature was 24.5 °C, blood pressure 145/70, heart rate 35 beats/minute, and respiratory rate 8 to 10 breaths/minute. Auscultation of his heart and lungs was unremarkable. He was unconscious with a Reaction Level Scale 85 (RLS-85) score of 4. RLS-85 gives a score between 1 and 8. An RLS-85 score of 4 indicates an unconscious patient who localizes but does not ward off when pain stimulated. He exhibited a slight anisocoria with his right pupil slightly bigger than his left.

The laboratory work-up at admission included moderate electrolyte disturbances and elevated liver enzymes. Drug and alcohol screens were negative.

**Fig. 1:**
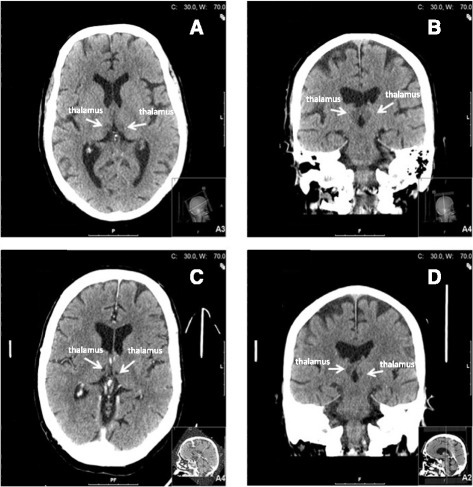
**a** Head computed tomography (axial) performed on the day of admission was initially assessed as normal, although signs of bilateral ischemia in the thalami actually were visible. **b** Head computed tomography (coronal) performed on the day of admission was initially assessed as normal, but later re-evaluated to be bilateral ischemia in the thalami. **c** Head computed tomography (axial) performed on day 24 of hospitalization showed bilateral ischemia in the medial areas of the thalami. **d** Head computed tomography (coronal) performed on day 24 of hospitalization showed bilateral ischemia in the medial areas of the thalami

An emergency CT of his head was performed and initially misinterpreted as normal with no signs of hemorrhage or acute infarction (Fig. 1a, b). In our intensive care unit (ICU), he was initially sedated and intubated with ventilator treatment. An attempt to extubate and wake him was made on day 3, but he still required ventilator support. He was re-intubated and sedated, and later given a tracheotomy. A complicated disease course followed with pneumothorax after CPR, pneumonia treated with antibiotics, bilateral pleural effusion requiring drainage, intestinal paralysis, acute pancreatitis, and ascites requiring paracentesis. The sedation was discontinued 2 weeks after admission to our ICU. He still required ventilation support. At this stage he could open his eyes when spoken to but otherwise he gave no contact. On neurological examination he withdrew his arms, moved his left foot, and grimaced upon pain stimulation. He had slight anisocoria, this time with his left pupil slightly bigger than his right. His pupillary reflexes were, however, symmetrical on direct and indirect stimulation. A follow-up CT performed on day 24 revealed bilateral ischemia in the medial areas of the thalami, as well as a smaller ischemic area in the left part of pons (Fig. 1c, d). At this point a senior neuroradiologist re-evaluated the first CT performed on admission and concluded that bilateral thalamic ischemia was discernable also on this CT (Fig. 1a, b). Electroencephalography (EEG) was pathological and showed an irregular theta/delta activity. However, there was no epileptiform activity.

**Fig. 2:**
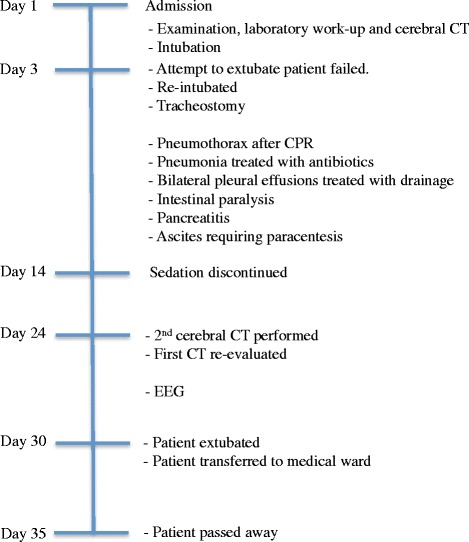
Timeline of events during the hospital stay. *CPR* cardiopulmonary resuscitation, *CT* computed tomography, *EEG* electroencephalography

Four weeks after admission it was possible to extubate him. His neurological function remained unchanged. He was transferred to our medical acute ward. On day 35 of hospitalization he died and the postmortem examination revealed pulmonary infarctions and pneumonia. A timeline of events is given in Fig. 2.

## Discussion

This case illustrates how the diagnosis of AOP infarct is often delayed preventing the correct treatment to be initiated. To the best of our knowledge this case is unique due to the special circumstances surrounding the patient, including his extremely low body temperature that caused him to develop a ventricular fibrillation requiring CPR in the ED upon admission. In addition, the initial head CT was evaluated as normal diverting focus away from the possibility of a cerebral incident.

With our patient being unconscious it was not possible to obtain a complete neurological status. Because his low body temperature could explain why he was unconscious and because the first CT was misinterpreted as normal, the correct diagnosis was initially missed. Contributing to this was the need for cardiopulmonary bypass treatment at admission to rewarm him due to his unstable heart function. The focus was therefore on cardiopulmonary issues, and not cerebral issues. Furthermore, since the first attempt to extubate him failed, he was kept sedated with ventilator support. Thus, the possibility of neurologically examining him while awake was again delayed. The slight anisocoria noted on admission was confounded by the fact that his pupils responded correctly to light both directly and indirectly. With a normal CT, no further action was taken.

The protracted clinical course that followed kept the physicians focused on the pulmonary and abdominal complications that developed. Thus, by the time he was stable and could be taken off sedation and ventilator support more than 3 weeks had passed. At this stage a neurological examination indicated cerebral pathology and a new CT was done in which the AOP infarct was diagnosed.

AOP infarction is not the only condition that can result in bilateral thalamic lesions. Other vascular etiologies of bilateral thalamic lesions include top of the basilar syndrome and deep cerebral venous thrombosis. Top of the basilar syndrome can present with bilateral thalamic infarcts but there are usually also infarcts present within the vascular territories of the superior cerebellar artery and posterior cerebral artery [18, 20]. Deep cerebral venous thrombosis can in rare instances result in bilateral symmetric involvement of the thalamus and basal ganglia [20, 21]. Wernicke’s encephalopathy can also be a differential diagnosis of bilateral thalamic lesions, in which T2-weighted MRI findings include symmetric hyperintensity in the medial thalami but also in the tectal plate, periaqueductal gray, mamillary bodies, and dorsal medulla [20, 21]. Differential diagnoses also include neoplasms, infections, Wilson’s disease, and osmotic myelinolysis [3, 20, 21].

There are several reports on AOP infarction in which initial CT was evaluated as normal [4, 6, 7, 22, 23]. This corresponds with the first CT in our case that was misinterpreted as normal. However, there are also reports on cases where initial MRI was unremarkable, which indicates that a normal initial MRI cannot exclude the diagnosis [23, 24]. Thus, in patients suspected to have an AOP occlusion, a repeat radiological examination may therefore be of value if the initial examination is normal.

The prognosis of thalamic infarcts is generally regarded as relatively good with regard to mortality and permanent motor deficits [1]. In a study, in which the long-term prognosis of 15 patients with AOP infarcts was investigated, a favorable outcome was defined as Modified Rankin Scale (mRS) score ≤2. In this study, 67% of patients with bilateral paramedian thalamic infarcts without midbrain involvement had a favorable outcome. By comparison, only 25% of patients with combined bilateral paramedian thalamic and rostral midbrain infarcts had a favorable outcome [17]. This suggests that the prognosis of AOP infarction is generally favorable, except when the midbrain is involved. The fact that our patient also had a small ischemic area in the pons could be one reason for the absence of improvement in his neurological functions.

## Conclusions

AOP infarcts are rare. Because the initial radiological assessment often is judged normal, repeated CT or MRI may be of value if AOP infarct is clinically suspected. The symptoms of AOP may also be variable depending on the size and distribution of the infarct. Thus, the clinical symptoms may also vary and in combination with the radiological challenges make this condition harder to diagnose. In addition, physicians are unfamiliar with AOP infarct diagnosis because it is infrequent. Case reports are therefore a valuable means to improve awareness of AOP infarcts.

## Abbreviations

AOP: Artery of Percheron
CPR: Cardiopulmonary resuscitation
CT: Computed tomography
DWI: Diffusion-weighted imaging
ED: Emergency department
EEG: Electroencephalography
FLAIR: Fluid-attenuated inversion recovery
ICU: Intensive care unit
MRI: Magnetic resonance imaging
mRS: Modified Rankin Scale
P1: Proximal segment of the posterior cerebral artery
RLS-85: Reaction Level Scale 85
SR: Sinus rhythm.
